# Mexico City Prospective Study: cohort profile update

**DOI:** 10.1136/bmjmed-2025-002223

**Published:** 2026-09-18

**Authors:** Jonathan Emberson, Jesus Alegre-Diaz, Jaime Berumen, Pablo Kuri-Morales, Roberto Tapia-Conyer

**Affiliations:** 1 Nuffield Department of Population Health, University of Oxford, Oxford OX3 7LF, UK; 2 Experimental Research Unit from the Faculty of Medicine, National Autonomous University of Mexico, Mexico City, Mexico; 3 Tecnológico de Monterrey, Monterrey, Mexico; 4 Faculty of Medicine, National Autonomous University of Mexico, Mexico City, Mexico

## Abstract

To fully understand the causes of human disease worldwide, large blood based prospective studies are needed in a range of populations with different healthcare settings and exposures to environmental and genetic risk factors. The Mexico City Prospective Study, updated in this cohort profile, was established in the late 1990s to investigate the sociodemographic, lifestyle, physical, and biological (including genetic) determinants of disease among around 150 000 adults in Mexico City. The study has over 20 years of mortality follow-up, cohort-wide biochemical and genetic data, repeat survey data from a subset of surviving participants, and ongoing ascertainment of non-fatal diseases. All Mexico City Prospective Study data are available for open access academic research within a trusted research environment, with preferential access for researchers based in Mexico and no data access fees for researchers in low income or middle income countries. Beyond representing a genetically diverse Latin American population, a key distinguishing feature of the study is the high baseline prevalence of obesity and diabetes. Earlier findings showed that diabetes had a substantially greater impact on mortality than suggested by evidence from high income countries, with a third of all deaths at ages 35-74 years attributed to diabetes. These results contributed to rapid changes in national disease prevention policies. By combining a genetically admixed (ie, individuals with ancestry from multiple ancestral populations) Latin American population, family based recruitment, a high burden of metabolic disease, availability of biochemical and genetic data, long term follow-up, and open access data availability, the Mexico City Prospective Study offers unique opportunities for health related discoveries within Mexico and internationally.

**KEY MESSAGES**

- The Mexico City Prospective Study is one of the longest and largest population based studies of a Latin American population
- The study includes sociodemographic, lifestyle, physical, biochemical, and genetic data from a highly admixed population
- National mortality linkage is complemented by house-to-house follow-up for non-fatal diseases
- The data generated are fully open access, with priority for researchers in Mexico and free access for researchers in low income and middle income countries

## Introduction

 The Mexico City Prospective Study is a large, long term, blood based cohort study of Mexican adults,^1^ established in the late 1990s by scientists at the Universidad Nacional Autónoma de México in a long running collaboration with scientists at the University of Oxford. The study was initially conceived to investigate the health effects of tobacco use in people in Mexico, but soon expanded into a broader prospective study designed to examine a wide range of risk factors, including those measurable in blood. The data are openly available to academic researchers worldwide,^2^ maximising their potential to improve human health.

## Study population

**Figure 1:**
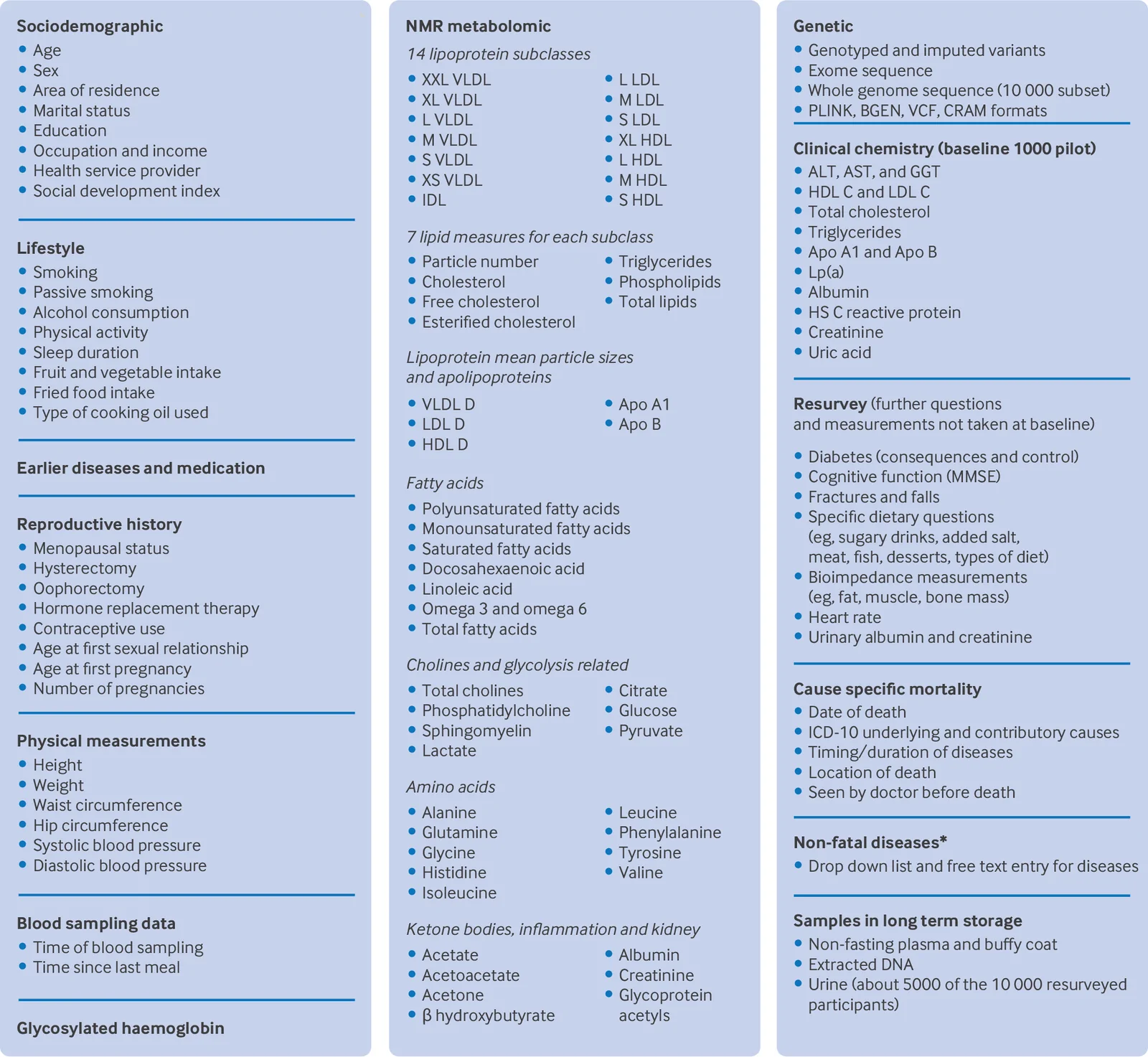
Types of data and samples currently available. Unless otherwise stated, the data/samples are available for nearly all participants at baseline and the 10 000 resurveyed participants.*Collected since 2019 through door-to-door interviews of survivors or suitable proxy respondents. ALT=alanine aminotransferase, Apo A1=apolipoprotein A1, Apo B=apolipoprotein B, AST=aspartate aminotransferase, C=cholesterol, D=diameter, GGT=gamma glutamyl transpeptidase, HDL=high density lipoprotein, IDL=intermediate density lipoprotein, L=large, LDL=low density lipoprotein, Lp(a)=lipoprotein(a), M=medium, MMSE=mini mental state examination. S=small, VLDL=very low density lipoprotein, XL=extra large, XXL=extra extra large, XS=extra small

The study is set in two contiguous urban districts (Coyoacán and Iztapalapa) of central and northeast Mexico City, populated by a diverse mix of long term residents and relatively recent migrants from the north and south of Mexico. Between April 1998 and September 2004, 107 111 women and 52 644 men aged 35 years or older (mean age 50 years) were recruited from their homes. Most study visits were predominantly made during standard working hours, however, visits were extended into the early evenings and at weekends in order to increase the proportion of men included in the study. Despite these efforts, recruitment remained higher among women than men. Of 112 333 households visited, one or more eligible individuals from 106 059 households agreed to take part (94% response). A summary of the data and samples available at each time point is provided in figure 1 and key baseline characteristics are provided in table 1.

**Table 1:** Selected baseline characteristics of the Mexico City Prospective Study by participant age and sex. Data are per cent, unless otherwise indicated

Characteristic	Participant age (years)						
	Men			Women			All*
	35-54	55-74	≥75	35-54	55-74	≥75	
No of participants	30 559	17 556	4464	66 994	32 328	7616	159 517
Mean age (years)	44	64	81	44	64	81	53
Median birth year	1957	1938	1922	1958	1939	1922	1951
Resident in Coyoacán	45	42	35	39	39	36	40
Completed high school or higher level of education†	32	13	7	16	4	2	15
Smoking behaviour:							
Never smoker	20	20	24	58	71	76	49
Former smoker	23	40	52	14	16	17	20
Current smoker	57	40	23	29	13	6	31
Regular alcohol drinker‡	36	27	16	7	5	4	15
Regular leisure time physical activity§	28	26	22	17	20	15	21
Physical measurements:							
Body mass index	28.1	27.9	26.4	29.5	29.9	27.6	29.0
Waist-hip ratio	0.94	0.97	0.97	0.87	0.90	0.93	0.90
SBP/DBP (mm Hg)	125/84	134/86	136/84	122/81	134/85	139/84	128/83
Earlier self-reported disease:							
Diabetes	9	22	18	8	25	21	14
Coronary heart disease	1	4	5	1	2	4	2
Stroke	1	2	4	1	2	3	1
Cancer	<0.5	1	2	1	2	2	1
Blood sample taken	97	97	97	98	97	96	97
Selected laboratory measurements							
HbA1c (%)¶	5.9	6.4	6.2	5.8	6.6	6.3	6.1
eGFR (mL/min/1.73m^2^)	104	88	76	105	87	74	97
LDL cholesterol (mmol/L)	2.42	2.34	2.17	2.45	2.56	2.39	2.44
HDL cholesterol (mmol/L)	0.93	0.93	0.93	1.03	1.04	1.04	1.00
Triglycerides (mmol/L)	1.69	1.61	1.35	1.50	1.63	1.45	1.57
Apolipoproten B (g/L)	0.89	0.88	0.83	0.88	0.93	0.89	0.89
Apolipoproten A1 (g/L)	1.17	1.16	1.12	1.24	1.26	1.24	1.22
Genetic data passing quality control:							
Genotype array	88	87	88	89	89	88	88
Exome sequence	88	87	88	89	89	88	88
Whole genome sequence	5	9	11	5	7	6	6
Has at least one first degree relative in the study	42	39	53	38	47	61	42
Mean inferred ancestry							
Indigenous American	69	67	66	69	68	66	68
European	27	29	30	27	28	30	28

Percentages may not add to 100 owing to rounding. Lipids (LDL cholesterol, HDL cholesterol, triglycerides, apolipoprotein A1, apolipoprotein B) measured using the Nightingale Health nuclear magnetic resonance platform.

* A small number of participants were recruited twice at different time points. For these participants, the data reflect the first time point at which a blood sample was taken.

† In Mexico, children typically graduate high school between ages 16 and 18 years of age.

‡ Drinking alcohol at least once a month.

§ At least one day of leisure-time physical activity per week.

¶ Unit of measurement for HbA1c (ie, not percentage of participants)

BMI, body mass index; DBP, diastolic blood pressure; eGFR, estimated glomerular filtration rate, calculated using CKD-EPI (2009) equation with creatinine measured by nuclear magnetic resonance; HbA1c, glycosylated haemoglobin; HDL, high density lipoprotein; LDL, low density lipoprotein; SBP, systolic blood pressure.

## Study visits and follow-up

All data collected through face-to-face interviews, linkage to the national mortality register, and assays of stored blood samples, can be viewed on the data showcase (https://datashare.ndph.ox.ac.uk/mexico/), which includes the exact study questions and data distributions (including extent of missing data).

### Baseline assessment (1998-2004)

Trained nurses conducted baseline visits following standardised procedures, with questionnaire answers recorded directly into hand held electronic devices. Collected data included sociodemographic information, lifestyle, reported medical history, and medication use. The nurses used calibrated electronic scales to measure weight, stadiometers to measure height, and non-stretchable tape to measure waist and hip circumference. Systolic and diastolic blood pressure when the participant was seated were measured three times (at one minute intervals) to the nearest 1 mm Hg using a standard mercury sphygmomanometer. Almost all participants (97%) provided a blood sample in a single 10 mL venous EDTA (ethylenediaminetetraacetic acid) vacutainer. Samples were placed in cool boxes, transferred to the study laboratory at the end of each day, refrigerated overnight at 4°C, then spun and separated the next working day. Three cryovials of plasma and one of the buffy coat containing DNA were separated, stored locally at −80 °C for a few months, then shipped to the UK on dry ice for long term storage in the Clinical Trial Service Unit and Epidemiological Studies Unit Wolfson laboratories in Oxford.

### Resurvey assessment in a subset of survivors (2015-19)

Between June 2015 and February 2019, 29 001 households of the originally recruited cohort were randomly selected and revisited, from which 8278 (29%) households provided a total of 10,143 participants who were still living at the same address, home at the time of the revisit, and agreed to take part in the resurvey. The primary purpose of the resurvey was to assess the extent of changes in risk factors over time and in particular the extent of within-person variability (to enable prospective analyses to be corrected for regression dilution bias).^3^ The resurvey also included more detailed questions and measurements (including bioimpedance estimated body composition measures, cognitive function, and diabetes treatment questions), which can be used to calibrate less precise measures conducted in the whole cohort at baseline. Resurvey blood samples were collected, processed, and stored similarly to the baseline assessment samples and, for a subset of about 5000 participants, a urine sample was also collected, frozen, and stored. The resurveyed participants were broadly representative of all participants who were still alive and living in the same location as when they were initially recruited.

### Follow-up for mortality

Deaths are identified through probabilistic matching (based on name, age, and sex) to the Mexican System for Epidemiologic Death Statistics (Subsistema Epidemiológico y Estadístico de Defunciones, SEED) electronic death registry in Mexico City, administered by the Ministry of Health. Field validation of more than 7000 matched deaths was done between 1998 and 2008, and confirmed the reliability of the algorithm in >95% of cases (ie, the positive predictive value was >95%). By mid-2022, about 35 000 (22%) participants had been matched to a death certificate using this algorithm.

## Recent study enhancements

### Cohort-wide assays of stored blood samples

Baseline and resurvey samples of plasma, buffy coat and, for a subset of resurveyed participants, urine are in long term storage in the UK. Cohort-wide assays of glycosylated haemoglobin were analysed from the buffy coat samples in Clinical Trial Service Unit and Epidemiological Studies Unit ISO17025-accredited Wolfson laboratories.^4^ In addition, assays of nuclear magnetic resonance metabolomic biomarkers were analysed in all baseline and resurvey samples using Nightingale Health's high throughput nuclear magnetic resonance metabolomics platform, providing information on 228 plasma biomarkers as absolute concentrations or ratios.^5^ DNA was extracted from the buffy coat samples and, through an industry academic partnership with Regeneron and AstraZeneca, cohort-wide genotyping (using version 2 of the Illumina global screening array) and exome sequencing (using the Illumina NovaSeq 6000 platform) was completed.^6^ Finally, through further collaboration with Abbvie, a subset of 10 008 participants have been whole genome sequenced using the Illumina NovaSeq 6000 platform (with 99% of samples having mean genome coverage >30x).^6^

### Health follow-up survey

Collection of information about non-fatal diseases can greatly enhance the ability of prospective studies to investigate a broad range of common and rare diseases. In 2019, interviewers began conducting door-to-door health follow-up surveys (similar to those used in many large scale prospective studies done in middle income countries where direct linkage to electronic health records is not possible). The survey includes information on major disease diagnoses received by participants, reasons for admission to hospital, and the year of each diagnosis or hospital admission. This survey is ongoing and, once completed, the data will be added to the data available for research.

## Data and sample availability

All Mexico City Prospective Study data are available to academic researchers for collaboration or open access data requests. We also welcome proposals from publicly and privately funded organisations for new assays of the stored biological samples (ie, plasma, buffy coat, and urine from resurveyed participants). The data and sample sharing policy is available from the study website^7^ and data variables can be examined through the online data showcase.^8^ The Mexico City Prospective Study allele frequencies that are specific to ancestry are available at: https://rgc-mcps.regeneron.com. A key feature of the data sharing policy is the preferential access given to researchers in Mexico for two years to newly released data, in an attempt to provide better equity rather than equality of access.^9^ Once projects are approved and a data transfer agreement is in place, access is provided through a DNAnexus research analysis platform (or, in exceptional circumstances for some non-genetics based research projects, through direct file transfer). A list of approved projects is provided on the study website.

## Study importance and earlier impact

**Figure 2:**
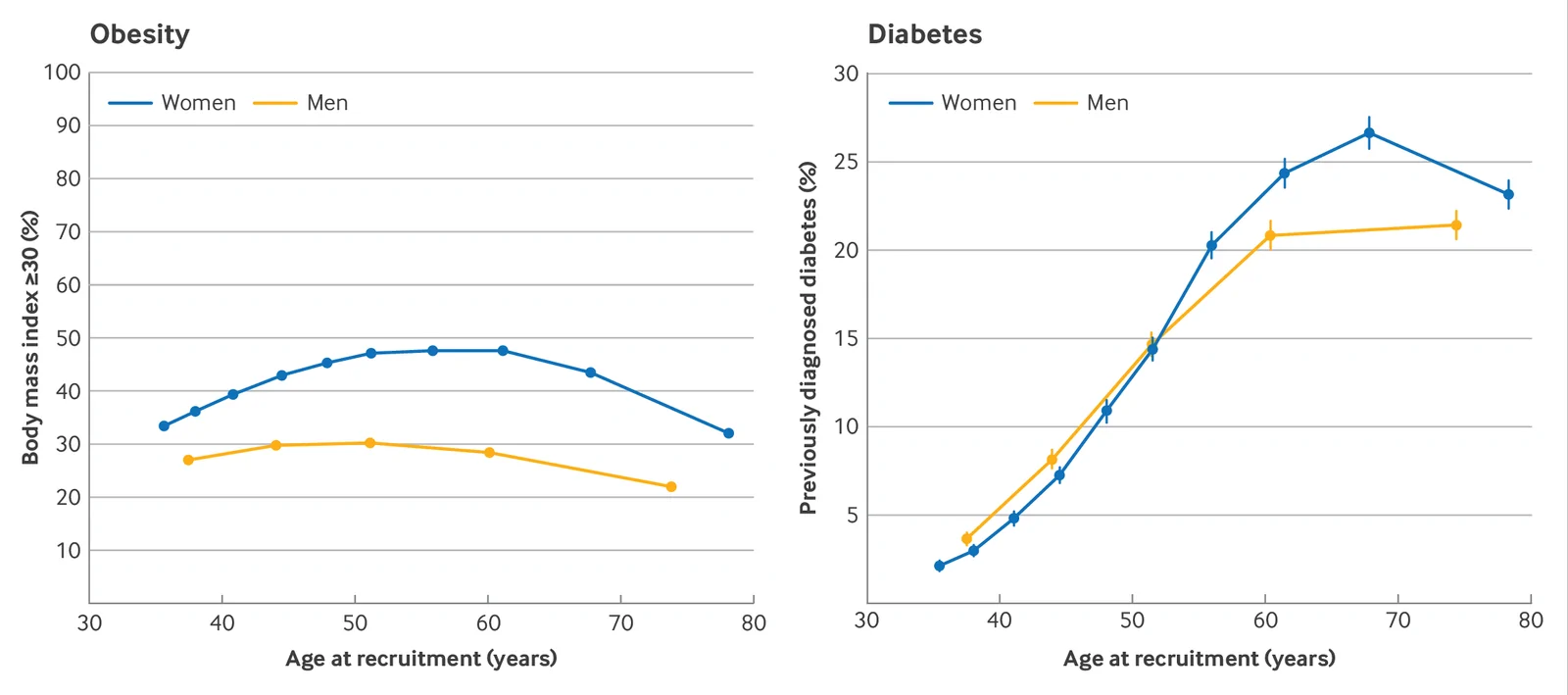
Prevalence of obesity (left panel) and previously diagnosed diabetes at participant recruitment (right panel), by age and sex. Adapted from Kuri-Morales et al.^11^ The figure denotes 10 groups for women and five for men, so that each group includes about 10 000 individuals

Large prospective blood-based studies in populations that differ in their socio-demographic, environmental, and genetic risk profiles (thereby extending the range of exposures studied) and disease incidence rates (thereby broadening the range of diseases that can be investigated) are needed to provide more widely generalisable evidence about the relevance of the full range of human exposures to many different health conditions worldwide.^10^ In this context, a key feature of the Mexico City Prospective Study, unique from most other large contemporary blood based cohorts, as well as from existing consortiums of blood based cohorts, is the extremely high prevalence of obesity and diabetes in participants at recuitment.^11^ Consistent with national estimates for Mexico around the same time,^12,13^ about half of the women and about one third of men aged 60 years at recruitment in the study had a body mass index of ≥30, while more than one in five had received a diagnosis of diabetes (figure 2). Over the past decade, the study has focused on evaluating the impact of these^14,15^ and other major disease risk factors (eg, blood pressure,^16^ smoking,^17^ alcohol consumption^18^) on premature death, resulting in several unexpected discoveries based on previous studies of high income populations or earlier, smaller studies of Latin American populations. A full list of study publications is available on the study website.^7^

**Figure 3:**
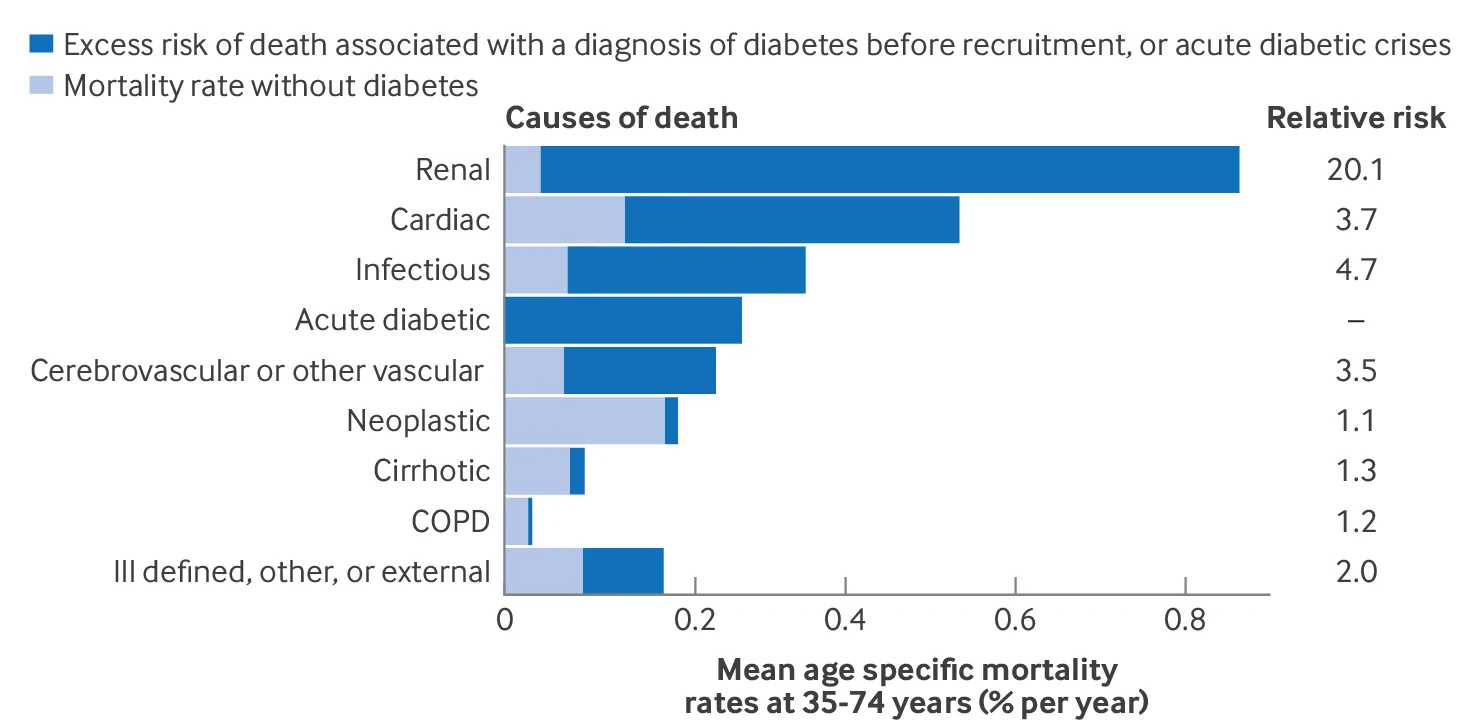
Mortality rate for specific causes of death in people with and people without a history of diabetes. Mortality rate in those without diabetes (light blue shading), the excess mortality in people who had received an earlier diagnosis of diabetes (dark blue shading) comprise the mortality rate in people with a history of diabetes (light plus dark blue bars). These mortality rates are age and sex standardised to 2012 death rates for Mexico. Infective diseases include peptic ulcer disease and exclude any infection in another plotted category. Numbers above the bars indicate rate ratio for participants with *v* without an earlier diagnosis of diabetes. COPD=chronic obstructive pulmonary disease. From: New England Journal of Medicine; Alegre-Díaz J, Herrington W, López-Cervantes M, Gnatiuc L, Ramirez R, Hill M, Baigent C, McCarthy MI, Lewington S, Collins R, Whitlock G, Tapia-Conyer R, Peto R, Kuri-Morales P, Emberson JR; Diabetes and Cause-Specific Mortality in Mexico City; 375; 1961-1971; Copyright © 2016 Massachusetts Medical Society. Reprinted with permission.

In particular, in 2016 we reported that people who had received earlier diagnoses of diabetes (a group that, on average, had very poor glycaemic control) had four times the death rate at ages 35-74 years compared with those who had not received diagnoses of diabetes (twice that expected based on evidence from previous studies of high income populations). Overall, one third of deaths were attributable to diabetes, with the largest absolute excess risk due to renal death (figure 3).^15^ These findings led to the introduction of national disease prevention policies targeting obesity and diabetes in Mexico.^19^ More recently, the Mexico City Prospective Study contributed to exome wide association studies that showed that the inhibition of GPR75 could provide a novel therapeutic strategy for obesity,^20^ that MAP3K15 is a potential future therapeutic target for diabetes,^21^ and that rare protein coding variants in CHRNB2 reduce the likelihood of smoking.^22^

## Strengths and limitations

Prospective cohort studies become increasingly more informative over time.^10^ With more than 150 000 recruited participants, more than 20 years of follow-up, and a wide range of environmental, physical, and biological measurements (including genetics), the chief strength of Mexico City Prospective Study is the ability to investigate the individual and joint contributions of genetic and non-genetic factors to a wide range of diseases in both middle and old age. The study also helps redress the prevailing bias in genomic research towards European populations by focusing on a Latin American population, enhancing the potential to discover novel risk variants that may occur at higher frequencies in this population. The family based design provides particular opportunities for genetic discovery by reducing or eliminating confounding caused by familial and demographic factors in analyses.^23^ Although the study includes participants from two districts of Mexico City only—so is not representative of Mexico nor Mexico City—prospective studies of non-representative cohorts can still provide reliable, widely generalisable evidence about the associations of risk factors with disease.^10^ Routine electronic health record linkage is restricted to mortality, and is based on a probabilistic algorithm based on name, age and sex. Consequently, some deaths may be missed, but this does not bias estimates of the relative effects of risk factors on causes of disease. Complete electronic linkage to non-fatal diseases is currently unavailable, but the ongoing door-to-door health follow-up surveys will allow future investigations of risk factors for these diseases by comparing self-reported and proxy reported cases with corresponding controls.

## Future plans

Key future priorities include continuing to build the scientific resource by performing additional assays (eg, proteomic or other omic assays) on the stored blood and urine samples, combining these data with ongoing follow-up of fatal and non-fatal events, and ensuring that these data remain equitably available to researchers worldwide.
