# Supplementary material for: Mexico City Prospective Study: cohort profile update
Source: BMJ Med. 2026 Sep 18;5(1):e002223. doi: 10.1136/bmjmed-2025-002223 (PMC13599889; doi:10.1136/bmjmed-2025-002223)
Supplement: Supplementary file 1 [file bmjmed-2025-002223.reviewer_comments.pdf]

PEER REVIEW HISTORY

BMJ Medicine publishes all reviews undertaken for accepted manuscripts. Reviewers are asked to complete a checklist review form and are provided with free text boxes to elaborate on their assessment. These free text comments are reproduced below.

ARTICLE DETAILS

**TITLE (PROVISIONAL)**	Cohort and Data Resource Update: Mexico City Prospective Study
**AUTHORS**	Emberson, Jonathan; Alegre-Diaz, Jesus; Berumen, Jaime; Kuri-Morales, Pablo; Tapia-Conyer, Roberto

VERSION 1 - REVIEW

**REVIEWER NAME**	Gete, Dereje G.
**REVIEWER AFFILIATION**	University of Queensland
**REVIEWER CONFLICT OF INTEREST**	None
**AI DISCLOSURE**	No
**DATE REVIEW RETURNED**	31-Oct-2025

**GENERAL COMMENTS**	This cohort and data resource update on the MCPS is well written. I have only a few minor comments and clarifications:<br>• The intro notes that the MCPS was established in the late 1990s, but it is unclear how many surveys have been conducted to date – only the baseline (1998–2004) and the resurvey (2015–2019)?<br>• Please clarify how mortality and disease outcomes have been continuously tracked since recruitment, including any data linkage methods used for follow-up.<br>• While baseline participant characteristics are described, details of the resurvey or follow-up are missing, which could be important for comparison, particularly regarding education. How was the limited representativeness addressed, given that participants were recruited from only two urban districts of Mexico City rather than a nationally representative sample?<br>• In the limitations section, could selection or survivor bias arise if participants who remained for follow-up differed from those lost or deceased?

**REVIEWER NAME**	Harron, Katie
**REVIEWER AFFILIATION**	
**REVIEWER CONFLICT OF INTEREST**	None
**AI DISCLOSURE**	No
**DATE REVIEW RETURNED**	03-Nov-2025

**GENERAL COMMENTS**	This is a clearly written data resource profile and I don't have any specific comments on how it could be improved.<br><br>On the figure, the labelling for the diabetes plot is obscuring the lines and should be redone.

**REVIEWER NAME**	Davies, Neil
**REVIEWER AFFILIATION**	UCL, Division of Psychiatry and Department of Statistical Science
**REVIEWER CONFLICT OF INTEREST**	None
**AI DISCLOSURE**	No
**DATE REVIEW RETURNED**	05-Nov-2025

**GENERAL COMMENTS**	This is an excellent profile for a leading cohort. It provides clear details about the information gathered by the study, and how the data were generated.<br><br>1. There are two remarkable features of MCPS, which in my view could be explained in more detail in the profile. First, the study has a household based design. As a result, it has sampled more biological relatives and family members than other study designs. This can be enormously useful for molecular genetic analyses, and perhaps provides a model for future studies.<br>2. Second, MCPS is a very unusual example of a very large genotyped study that has sampled from non-European populations. This touches on the strengths and limitations, but more details could be provided – for example, a box detailing the relatedness in the study – how many of each type of relative do you have? This is touched on in the section on how MCPS differs, but this could be explained in far more detail, quite what a major advantage this is. Not only do the health burden and needs of the population in Mexico differ, but insights about the mechanisms driving health differences in Mexico (especially genetics) can be relevant and actionable in all populations. This makes MCPS extremely valuable and distinctive, relative to other cohorts which merely provide increased samples sizes of populations that are very well characterised.<br>3. For the genetic data, it would be useful to provide full details of this. Are the array data imputed, what's the call rate, imputation reference panel, mix of ancestries, what's the storage format?<br>4. Could you provide a summary, e.g. in a Box, table or figure, of the stored samples, how many are still available, how many have multiple longitudinal samples, what type are they (e.g. serum, plasma, urine etc). What's the process for applying for using these samples – is this solely led by the PIs of the study, or are you open to public or privately funded efforts to get more proteomics or other assays for your cohort?<br>5. Could you provide any details on the relative representativeness of this sample versus the region and Mexico more broadly?<br>6. Could you provide any details about the number of studies that have been approved and papers that have used the study?<br>7. Could you add a figure that illustrates what was measured at each time, for example, something similar to Figure 1 in Shireby et al. (2025). This helps provide a visual summary for researchers interested in using the data:<br><br>https://academic.oup.com/ije/article/54/5/dyaf141/8237599

**REVIEWER NAME**	Islam, Nazrul
**REVIEWER AFFILIATION**	University of Southampton, School of Primary Care, Population Sciences and Medical Education
**REVIEWER CONFLICT OF INTEREST**	None
**AI DISCLOSURE**	No

DATE REVIEW RETURNED	10-Nov-2025

GENERAL COMMENTS	Thank you for the opportunity to review the manuscript which is written well. I hope the authors will find the following comments and suggestions useful for their revision.<br><br>One of the other limitations might be that the whole sample is from urban areas, so the findings may not be generalisable to rural areas. The male participants are also heavily underrepresented.<br>Could the authors provide a head-to-head comparison of some of the estimates (eg., obesity, diabetes) with those from the national (or regional) estimates of Mexico. This would give us more insights about the type of participants in the sample.<br><br>While the relative measures may not be affected, the absolute measures might. Hence, a cautionary note that absolute measures such as the prevalence/incidence risk/rates may not be taken as the risk/rates. Also, sometimes the rate/risk differences are also considered as relative measures. However, while the risk/rate ratio may be less affected, the rate/risk differences might be more so.<br><br>Overall, that the risk/rate ratio will be less affected is based on the assumption that the measurement errors are non-differential among the comparison groups (eg, among those with and without diabetes, in the example), but not sure if that is a reasonable assumption or sometimes not so much.<br><br>The figure is not entirely clear to me. The white boxes refer to the rate without diabetes, but the shaded ones are ‘excess risk’? What is the scale of that? Risk difference? I suggest showing them side-by-side (rather than stacked) to show the absolute rates, and the RR column to show the rate ratio (as it is now).<br><br>Could the authors include a section on missing data.<br><br>Could the authors provide the periodicity of the data collection (how many waves, how often etc).<br>A visual flow diagram showing how many participants continued to remain in the cohort, and how many participants were later included (and whether they remained or lost to follow-up) including their children could be very informative.<br><br>Could the authors provide a data dictionary including the definitions of the covariates/features, maybe as a supplement to make the paper more standalone/independent.

REVIEWER NAME	de Jesús Medina-Arellano, María
REVIEWER AFFILIATION	National Autonomous University of Mexico (UNAM), Instituto de Investigaciones Jurídicas
REVIEWER CONFLICT OF INTEREST	None
AI DISCLOSURE	No
DATE REVIEW RETURNED	03-Dec-2025

GENERAL COMMENTS	Thank you for the opportunity to review this well written and informative cohort update of the Mexico City Prospective Study (MCPS). The manuscript clearly describes major advances since the

	original cohort profile and effectively communicates the scientific value of this long standing collaboration. The update is timely, the writing is clear, and the study's potential for advancing research in populations that are typically under represented in large blood-based cohorts is evident.<br>The manuscript clearly lists approvals, but a short reflection on ethical considerations, particularly around broad consent for long term genetic and multi-omic analyses, would add depth.<br><br>Since MCPS began before contemporary standards for genomic governance were widely adopted, readers may appreciate clarification of how the study ensures ongoing ethical oversight and participant protection.<br>I woud like to provide minor comment to be addressed: Some URLs in the references are long, e.g. replacing them with DOI links where possible would improve formatting.<br>This is a strong and valuable cohort update. The study represents a uniquely important scientific resource, and the manuscript is well positioned for publication.

VERSION 1 – AUTHOR RESPONSE

Reviewer 1: Dr. Dereje G. Gete, University of Queensland

This cohort and data resource update on the MCPS is well written. I have only a few minor comments and clarifications:

• The intro notes that the MCPS was established in the late 1990s, but it is unclear how many surveys have been conducted to date – only the baseline (1998–2004) and the resurvey (2015–2019)?

> *Response:* The revised 'Study visits and follow-up' section (line 111) now makes it clearer that the surveys included only the baseline assessment and the partial resurvey, with follow-up for mortality done through passive linkage, and information on non-fatal diseases collected through current fieldwork (see 'Recent study enhancements' section [line 157])

• Please clarify how mortality and disease outcomes have been continuously tracked since recruitment, including any data linkage methods used for follow-up.

> *Response:* Details provided on lines 148-55 for mortality and 190-200 for morbidity.

• While baseline participant characteristics are described, details of the resurvey or follow-up are missing, which could be important for comparison, particularly regarding education. How was the limited representativeness addressed, given that participants were recruited from only two urban districts of Mexico City rather than a nationally representative sample?

> *Response:* Details of the resurvey and follow-up procedures are provided on lines 132-46 and 148-55 respectively. Characteristics of the resurveyed participants are provided in the online Data Showcase (line 208-9). The study was never designed to be representative of adults in Mexico, Mexico City, or even the two study districts. However, by including a large number of men and women who vary with respect to socio-demographic, lifestyle, physiological and genetic factors, the study is able to generate lasting evidence about the associations of particular risk factors with health outcomes that are likely to be widely generalisable (lines 260 -4).

• In the limitations section, could selection or survivor bias arise if participants who remained for follow-up differed from those lost or deceased?

Response: The resurveyed participants were broadly representative of all participants who were still alive and living in the same house as the time they were originally recruited (now stated on line 144-6).

<u>Reviewer 2: Prof. Katie Harron</u>

This is a clearly written data resource profile and I don't have any specific comments on how it could be improved.

On the figure, the labelling for the diabetes plot is obscuring the lines and should be redone.

Response: Thank you. The plot has now been fixed.

<u>Reviewer 3: Prof. Neil Davies, UCL</u>

This is an excellent profile for a leading cohort. It provides clear details about the information gathered by the study, and how the data were generated.

1. There are two remarkable features of MCPS, which in my view could be explained in more detail in the profile. First, the study has a household based design. As a result, it has sampled more biological relatives and family members than other study designs. This can be enormously useful for molecular genetic analyses, and perhaps provides a model for future studies.

Response: This strength of the study has now been highlighted on lines 258-60. The Table now includes information on the number of people with a first degree relative in the study.

2. Second, MCPS is a very unusual example of a very large genotyped study that has sampled from non-European populations. This touches on the strengths and limitations, but more details could be provided – for example, a box detailing the relatedness in the study – how many of each type of relative do you have? This is touched on in the section on how MCPS differs, but this could be explained in far more detail, quite what a major advantage this is. Not only do the health burden and needs of the population in Mexico differ, but insights about the mechanisms driving health differences in Mexico (especially genetics) can be relevant and actionable in all populations. This makes MCPS extremely valuable and distinctive, relative to other cohorts which merely provide increased samples sizes of populations that are very well characterised.

Response: Due to restrictions on the number of non-text items, we have included further information on genetic ancestry and relatedness in the Table. We have also elaborated on lines 258-60 how such features provide particular research opportunities that are not available in Euro-centric studies.

3. For the genetic data, it would be useful to provide full details of this. Are the array data imputed, what's the call rate, imputation reference panel, mix of ancestries, what's the storage format?

Response: Full details of the genetic data available are provided in our genetics profile paper published in *Nature* (reference 6). In the current article (lines 174-88), we have described at a fairly high level that this data includes cohort-wide genotyping (using version 2 of the Illumina Global Screening Array), exome sequencing (using the Illumina NovaSeq 6000 platform) and, in a subset of 10,008 participants, whole genome sequencing. We also refer the reader to the online variant browser where ancestry-specific allele frequencies can be viewed (lines 209-

10), the website where storage formats can be viewed (line 216), and have added further information on genetic ancestry to the Table.

4. Could you provide a summary, e.g. in a Box, table or figure, of the stored samples, how many are still available, how many have multiple longitudinal samples, what type are they (e.g. serum, plasma, urine etc). What's the process for applying for using these samples – is this solely led by the PIs of the study, or are you open to public or privately funded efforts to get more proteomics or other assays for your cohort?

Response: We have added further information regarding sample availability and the process for applying to access such samples (which can be from public or privately funded organisations) on lines 202-16.

5. Could you provide any details on the relative representativeness of this sample versus the region and Mexico more broadly?

Response: As mentioned in a reply to reviewer 1, the study was never meant to be representative of adults in Mexico, Mexico City, or even the two study districts. However, by including a large number of men and women who vary with respect to socio-demographic, lifestyle, physiological and genetic factors, the study is able to generate lasting evidence about the associations of particular risk factors with health outcomes that are likely to be widely generalisable (lines 260-4 and reference 8).

6. Could you provide any details about the number of studies that have been approved and papers that have used the study?

Response: This information is provided on the study website (lines 215-6).

7. Could you add a figure that illustrates what was measured at each time, for example, something similar to Figure 1 in Shireby et al. (2025). This helps provide a visual summary for researchers interested in using the data:

https://academic.oup.com/ije/article/54/5/dyaf141/8237599

Response: We have provided a new Box describing the types of samples and data currently available in the study.

Reviewer 4: Dr. Nazrul Islam, University of Southampton

Thank you for the opportunity to review the manuscript which is written well. I hope the authors will find the following comments and suggestions useful for their revision.

One of the other limitations might be that the whole sample is from urban areas, so the findings may not be generalisable to rural areas. The male participants are also heavily underrepresented.

Response: We agree but, as mentioned in a reply to reviewers 1 and 3, the study was never designed to be representative of adults in Mexico, Mexico City, or even the two study districts. However, by including a large number of men and women who vary with respect to socio-demographic, lifestyle, physiological and genetic factors, the study is able to generate lasting evidence about the associations of particular risk factors with health outcomes that are likely to be widely generalisable (lines 260-4 and reference 8).

The lower proportion of men than women in the study reflects the difficulties faced in recruiting men from their homes during the main recruitment times (ie, week days during working hours, as mentioned on lines 102-5). However, the study still includes 50,000 men

and 100,000 women so in many cases is able to provide reliable estimates of associations separately in men and women.

Could the authors provide a head-to-head comparison of some of the estimates (eg., obesity, diabetes) with those from the national (or regional) estimates of Mexico. This would give us more insights about the type of participants in the sample.

Response: Now stated on line 227 that baseline estimates of the prevalence of obesity are consistent with national estimates around the same time.

While the relative measures may not be affected, the absolute measures might. Hence, a cautionary note that absolute measures such as the prevalence/incidence risk/rates may not be taken as the risk/rates. Also, sometimes the rate/risk differences are also considered as relative measures. However, while the risk/rate ratio may be less affected, the rate/risk differences might be more so.

Response: We agree (as noted on lines 268-9), and would always recommend that researchers analysing data from MCPS (or other prospective studies) apply such caution when interpreting observed absolute risk or rate differences estimated within a study.

Overall, that the risk/rate ratio will be less affected is based on the assumption that the measurement errors are non-differential among the comparison groups (eg, among those with and without diabetes, in the example), but not sure if that is a reasonable assumption or sometimes not so much.

Response: We agree (the original paper [reference 13] discusses this question).

The figure is not entirely clear to me. The white boxes refer to the rate without diabetes, but the shaded ones are 'excess risk'? What is the scale of that? Risk difference? I suggest showing them side-by-side (rather than stacked) to show the absolute rates, and the RR column to show the rate ratio (as it is now).

Response: The figure on the right hand side is adapted from a previous publication (reference 13) and shows: a) the mortality rate in those without diabetes (white boxes); b) the excess mortality in those with diabetes [grey]; and, hence, c) the mortality rate in those with diabetes (white + grey). The legend to the figure has been revised to explain this.

Could the authors include a section on missing data.

Response: The best and most complete source of information on missing data is provided by our online Data Showcase (now stated on lines 112-5).

Could the authors provide the periodicity of the data collection (how many waves, how often etc).

Response: Now clarified in the new 'Study visits and follow-up' and 'Recent study enhancements' sections that the only face-to-face data collection periods were the original baseline assessment, the partial resurvey of a subset of participants an average of 15 years later, and the ongoing collection of information on non-fatal diseases.

A visual flow diagram showing how many participants continued to remain in the cohort, and how many participants were later included (and whether they remained or lost to follow-up) including their children could be very informative.

Response: The study never sought to recruit new participants after completion of the original baseline assessment. Information on the number of participants with at least 1 first degree relative in the study has been added to the Table.

Could the authors provide a data dictionary including the definitions of the covariates/features, maybe as a supplement to make the paper more standalone/independent.

> *Response:* The online Data Showcase (now first mentioned on lines 112-5) provides this information, including distributions of all variables collected. There is also an online variant browser providing variant allele frequencies (overall and by ancestry, line 209-10).

Reviewer 5: Dr. María de Jesús Medina-Arellano, National Autonomous University of Mexico (UNAM)

Thank you for the opportunity to review this well written and informative cohort update of the Mexico City Prospective Study (MCPS). The manuscript clearly describes major advances since the original cohort profile and effectively communicates the scientific value of this long standing collaboration. The update is timely, the writing is clear, and the study's potential for advancing research in populations that are typically under represented in large blood-based cohorts is evident.

The manuscript clearly lists approvals, but a short reflection on ethical considerations, particularly around broad consent for long term genetic and multi-omic analyses, would add depth.

> *Response:* Information on the consent procedure as related to future use of samples (including genetics) is provided on lines 319-23.

Since MCPS began before contemporary standards for genomic governance were widely adopted, readers may appreciate clarification of how the study ensures ongoing ethical oversight and participant protection.

> *Response:* We now state on 326-34 that study oversight is provided by the study principal investigators in Mexico and the UK, with ongoing relevant ethics approval in both countries, and a website that includes relevant information for participants.

I would like to provide minor comment to be addressed: Some URLs in the references are long, e.g. replacing them with DOI links where possible would improve formatting.
This is a strong and valuable cohort update. The study represents a uniquely important scientific resource, and the manuscript is well positioned for publication.

> *Response:* If accepted for publication we will work with the journal on the exact formatting for references and URLs.
